# Sleep Apnoea and Memory (SAM): protocol for a prospective study of prevalence and symptoms of sleep apnoea in memory clinics

**DOI:** 10.3389/fnagi.2026.1862599

**Published:** 2026-07-17

**Authors:** Victoria Grace Gabb, Charlotte Neary, Devan Mair, Adrian Kendrick, Gregor Russell, Julie Clayton, Samina Begum, Robert T. R. Huckstepp, Nicholas Turner, Elizabeth Coulthard

**Affiliations:** 1 Bristol Medical School, Faculty of Health and Life Sciences, University of Bristol, Bristol, United Kingdom; 2 ReMemBr Group, North Bristol NHS Trust, Bristol, United Kingdom; 3 NIHR Bristol Biomedical Research Centre, Bristol, United Kingdom; 4 School of Biosciences, Cardiff University, Cardiff, United Kingdom; 5 School of Applied Sciences, University of the West of England, Bristol, United Kingdom; 6 Department of Applied Dementia Studies, University of Bradford, Bradford, United Kingdom; 7 Research and Knowledge Service, Bradford District Care NHS Foundation Trust, Bradford, United Kingdom; 8 Independent Practitioner, Bradford, United Kingdom; 9 School of Life Sciences, University of Warwick, Coventry, United Kingdom

**Keywords:** Alzheimer’s disease, dementia, home sleep apnoea test (HSAT), memory, mild cognitive impairment, obstructive sleep apnoea (OSA), prevalence, sleep apnoea

## Abstract

**Introduction:**

Sleep apnoea is common in older adults and a risk factor for cognitive decline and dementia but is rarely assessed in memory clinics. The Sleep Apnoea and Memory (SAM) study will assess the prevalence of sleep apnoea and identify optimal screening for sleep apnoea in memory clinics.

**Methods:**

SAM is a prospective observational multi-site study recruiting adults attending NHS memory clinics. Participants will undergo a single night of polygraphy using a home sleep apnoea test (WatchPAT^®^ 300) and complete questionnaires based on NICE guidance for sleep apnoea assessment. The primary outcome will be the prevalence of sleep apnoea. Secondary outcomes include determining sleep apnoea prevalence across different cognitive diagnoses, identifying which symptoms and risk factors which best predict sleep apnoea, and assessing feasibility of remote sleep apnoea screening.

**Discussion:**

The SAM study will improve understanding of the extent of sleep apnoea in people attending memory clinics and inform design of an interventional trial for treating sleep apnoea in patients with cognitive impairment. Treating sleep apnoea in memory clinics may help to improve symptoms and/or prognosis for people experiencing memory problems.

## 1 Introduction

Sleep apnoea is a common sleep-related breathing disorder, particularly in older adults, in which breathing is frequently interrupted during sleep through complete pauses in breathing (apnoeas) and periods of slower or more shallow breathing (hypopneas) (Ghavami et al., 2023). Patients with sleep apnoea may present with daytime symptoms, such as daytime sleepiness or headaches, nighttime symptoms such as disturbed sleep and snoring, or may report no symptoms (Lacasse et al., 2002).

Sleep apnoea can broadly be categorised into obstructive sleep apnoea (OSA) and central sleep apnoea (CSA), though patients may present with both types (Zhang et al., 2023). OSA, where there is respiratory effort but no or partial airflow due to an upper airway blockage, is the most common type of sleep apnoea (Benjafield et al., 2019; Jordan et al., 2014). CSA, where both airflow and respiratory effort are reduced, is less common and typically results from comorbidities such as heart failure or stroke, or a side effect of medications (Randerath et al., 2024). Pauses in respiration result in recurrent intermittent hypoxia and hypercapnia, sleep fragmentation, increased sympathetic activity, and oxidative stress (Henning and Anderson, 2025; Bahia and Pereira, 2015). It is therefore unsurprising that sleep apnoea is associated with increased risk of cardiovascular disease, stroke, metabolic dysfunction, accidents, and mortality (Knauert et al., 2015). Despite advances in diagnosis and treatment of sleep apnoea, a high proportion of cases remain undiagnosed or ineffectively managed (Iannella et al., 2025).

The prevalence of sleep apnoea increases with age, with 1 in 7 adults (Benjafield et al., 2019) and 1 in 3 older adults (Ghavami et al., 2023) estimated to have sleep apnoea. Ageing is associated with various changes to sleep which could trigger or exacerbate sleep apnoea. During aging, sleep becomes more fragmented and increased arousal frequency can destabilise breathing, whilst changes to the upper airway, such as lengthening of the pharyngeal airway and reduced response to negative pressure, can predispose it to collapse (Glasser et al., 2011). Whilst sleep apnoea is more prevalent in men than premenopausal women, the prevalence of sleep apnoea in women increases markedly during and after menopause, likely influenced by changes to sex hormones and body composition (Jehan et al., 2016; Mirer et al., 2017). Additionally, older adults have a higher risk of multiple comorbidities hypothesised to have a bidirectional relationship with sleep apnoea, including diabetes (Muraki et al., 2018) and cardiovascular disease (Yeghiazarians et al., 2021; Glasser et al., 2011).

Sleep apnoea is also increasingly recognised as a potentially modifiable risk factor for cognitive decline and dementia (Lajoie et al., 2020; Gurbani et al., 2025; Ercolano et al., 2024). Untreated sleep apnoea has been associated with deficits in memory, attention, and executive function (Bucks et al., 2013) and patients with symptomatic OSA are more likely to develop mild cognitive impairment (MCI) or Alzheimer’s disease (AD; Bubu et al., 2020; Tsai et al., 2020). Elevated levels of several AD fluid biomarkers including amyloid-beta (Aβ) and tau pathology, oxidative stress, and inflammation are associated with sleep apnoea (Baril et al., 2018). Treating sleep apnoea can improve sleep and quality of life both in patients and their bed partners (Siccoli et al., 2008; Labarca et al., 2020; Fietze et al., 2023; Luyster, 2017), is associated with lower cerebrospinal fluid (CSF) Aβ burden (Ju et al., 2019), and can improve cognitive outcomes (Benkirane et al., 2024; Costa et al., 2023). Treatment can also have socioeconomic benefits through reducing healthcare utilisation costs and risk of accidents and improving productivity (Watson, 2016; Zappalà et al., 2025). Effective treatments for sleep apnoea include positive airway pressure (PAP), oral appliances, weight loss, hypoglossal nerve stimulation, and surgery to enlarge the upper airway (Gottlieb and Punjabi, 2020), though continuous PAP (CPAP) is often considered the first line treatment.

In recent years, there has been increasing recognition that screening for sleep apnoea in patients presenting with cognitive impairment is important (Liguori, 2025). For example, both the Canadian Consensus Conferences on the Diagnosis and Treatment of Dementia (Ismail et al., 2020) and Sleep Study Group of the Italian Dementia Research Association (Guarnieri et al., 2014) recommend that patients at risk for dementia should be screened for symptoms of sleep apnoea.

Identifying and treating sleep apnoea in memory clinics may therefore represent an opportunity to improve quality of life and cognitive and health outcomes. However, it is not yet understood how prevalent sleep apnoea is in patients presenting with cognitive symptoms, as sleep is rarely assessed in memory clinics. One retrospective cohort study in the Netherlands reported that 17% of their patients were diagnosed with OSA, with patients evenly split between those with pre-existing diagnoses and those diagnosed as a result of their memory clinic evaluation (Linssen et al., 2021). In contrast, a recent study conducted at a memory clinic in Australia identified OSA in 75% of their memory clinic patients, with only 13% previously having a diagnosis (Lam et al., 2025b). Beyond different prevalence estimates, these two studies also identified diverging relationships between OSA and severity of cognitive impairment. In the Netherlands memory clinic, prevalence was higher among patients with subjective cognitive decline compared to those with dementia (Linssen et al., 2021), whereas in the Australian memory clinic, patients with OSA had worse global cognition and memory performance compared to memory clinic patients without OSA (Lam et al., 2025b). Therefore, further research into prevalence in the memory clinic population and across different cognitive diagnoses is warranted.

Differences in the screening and diagnosis of sleep apnoea may partly underlie differences in prevalence estimates. For example, a systematic review into sleep apnoea prevalence in participants with MCI found that whilst prevalence estimates overall were 27%, prevalence estimates ranged significantly from 11% based on self-report to 71% based on polysomnography (PSG; Mubashir et al., 2019). Validated questionnaires such as the STOP-Bang (Chung et al., 2016) (designed to assess risk of sleep apnoea) and Epworth Sleepiness Scale (Johns, 1991; ESS; designed to assess daytime sleepiness) are often used to identify individuals at high risk of sleep apnoea who should undergo oximetry or polygraphy, including in primary care and perioperative settings (Rosenthal and Dolan, 2008; Senaratna et al., 2019). However, their predictive performance is limited, particularly in older adults and memory clinic populations (Lam et al., 2025a; Martins et al., 2020; Jorge et al., 2019). The ESS is also designed to assess sleepiness and is specifically not recommended to determine whether sleep apnoea assessment is warranted (NICE, 2021). Older adults typically present with fewer sleep apnoea symptoms compared to younger adults, and the commonest symptoms in older adults (such as sleepiness, memory loss, nocturia, and sexual dysfunction) may be attributed to ageing and/or neurodegeneration (Antonaglia et al., 2025). Therefore, objective data collection and identifying the most appropriate questionnaires for older adults and individuals with cognitive impairment is warranted.

In adult populations, including older adults, in-laboratory PSG is considered the gold standard (or Type I) test for sleep apnoea diagnosis in older adults. PSG collects data across many channels including an electroencephalogram (EEG), electrooculogram, electromyogram, and electrocardiogram (ECG) (Table 1). However, PSG requires significant resources and can be burdensome for patients, meaning it is impractical to use for large-scale screening. Home sleep apnoea tests (HSATs) or Type III respiratory polygraphy devices have the potential to improve access to sleep apnoea diagnosis at scale, without requiring a visit to a sleep clinic or other healthcare provider (Riha et al., 2023). Several HSATs have been approved for use by the Food and Drug Administration (FDA) and National Institute for Health and Care Excellence (NICE) in diagnosing sleep apnoea, including the WatchPAT^®^ and AcuPebble™ (Park et al., 2024; NICE, 2024).

**TABLE 1:** Sleep studies used in sleep apnoea screening and diagnosis: type I, II, III, and IV.

Type	Type of measurement	Attended or unattended	Example of measures collected
I	Polysomnography (PSG)	Attended, typically in a dedicated sleep laboratory in a hospital	Typically includes electroencephalography (EEG), electro-oculography (EOG), electromyography (EMG), electrocardiography (ECG), airflow, thoracoabdominal movement, snoring, body position, oxygen saturation.
II	PSG	Unattended	Same as PSG but unsupervised.
III	Home sleep apnoea tests (HSATs); polygraphy	Unattended	2 + respiratory variables, oxygen saturation, and 1 + cardiac variable
IV	Limited channel studies	Unattended	1 or 2 parameters; often oxygen saturation or airflow.

### 1.1 Study objectives

Our broad objective is to work toward future clinical trials treating sleep apnoea in memory clinics to see if treatment slows progression of neurodegeneration and/or improves patients’ quality of life. The present study aims to find out the prevalence, symptoms and risk factors of sleep apnoea, feasibility of remote sleep apnoea testing, and whether sleep apnoea treatment may improve blood biomarkers relating to AD and neurodegeneration in patients attending memory clinics.

Specifically, the study will:

(1) Determine the proportion of people attending memory clinics who have at least mild sleep apnoea, as defined by an oxygen desaturation index (ODI) 3% of ≥5, and whether prevalence differs by cognitive diagnosis.
(2) Identify the symptoms or risk factors that best predict sleep apnoea in people who present to memory clinics.
(3) Assess the feasibility of remote questionnaires for sleep apnoea screening in memory clinics.
(4) Pilot methodology for observing the effect of treating sleep apnoea on blood biomarkers of AD and neurodegeneration.

## 2 Methods and analysis

This is a pragmatic, prospective, observational, multi-site study designed to assess the prevalence and predictors of sleep apnoea in memory clinic patients in England. The study is sponsored by North Bristol NHS Trust and funded by the National Institute of Health and Care Research (NIHR) Research for Patient Benefit programme (NIHR207185). The study was prospectively registered on the International Standardised Randomised Controlled Trial Number (ISRCTN) registry (reference: ISRCTN11309942). The expected recruitment period is 10 months from the first site opening.

### 2.1 Ethics and informed consent

The study received a favorable opinion from the Health Research Authority and North West - Greater Manchester South Research Ethics (reference: 25/NW/0221) and will be conducted in line with Good Clinical Practice, the Declaration of Helsinki, and the Mental Capacity Act.

Prospective participants will be provided with a participant information sheet to explain the purpose of the study, the study procedures, and implications for the patient if they are found to experience daytime sleepiness or are diagnosed with sleep apnoea. Prior to consent, the study will be explained by a member of the research team, and patients will be given time to consider whether they would like to participate and the opportunity to ask questions and discuss their involvement with others, such as partners or relatives.

Where individuals have capacity to consent to the study, written informed consent will be obtained prior to participation. Where individuals do not have capacity to consent to the study, the research team will seek to identify an appropriate consultee who can advise on the patient’s likely wishes and feelings regarding study participation and any advance decisions made. The patient will be involved as fully as possible in any discussions around participation and signs of objection or dissent will trigger study withdrawal.

### 2.2 Participants

#### 2.2.1 Recruitment

Eligible participants will be adults aged 18 or over attending memory or cognitive disorders services at participating NHS sites (in Bristol, London, Cornwall, Northumbria, and Bradford) or affiliated participant identification centers in the 6 months prior to the date of consent. Patients may be approached prior to or during their clinical appointment, or within 6 months of an appointment. Appointments may be initial consultations or follow-up appointments, and patients will not be excluded based on any diagnosis. As the primary aim is estimating sleep apnoea prevalence, individuals with an existing diagnosis of sleep apnoea will also be eligible for inclusion and will be asked to continue their usual treatment for the duration of the study.

#### 2.2.2 Sample size

With prevalence based on objective measures in similar populations ranging from 17% (Linssen et al., 2021; Mubashir et al., 2019) to 75% (Lam et al., 2025b), a sample size of 385 participants was chosen to allow for estimation of 50% prevalence with a high degree of precision. The proposed sample size will also allow investigation of 16 parameters as predictors for sleep apnoea, balances accuracy with model overfitting, and will ensure that predictions will have a small error in the estimated outcome probabilities in the population (as measured by a mean absolute prediction error of 0.08) and that there is a small difference in performance in the data used for development and in the target population (difference in model R2 in development data and target population of 0.05).

It is anticipated that some participants may be unable to operate or tolerate the WatchPAT^®^ device for the minimum duration required for analysis (4 h) or may not complete the questionnaires. In our recent feasibility study using an overnight oximetry device to screen for sleep apnoea, at least one night of usable data was obtained from 33/37 (89%) participants: 3 (8%) participants declined to use the device and 1 (3%) found the device too uncomfortable to sleep (Gabb et al., 2025). To account for this, the overall recruitment target is 453 participants, with complete data from 385 participants, which allows for a 15% drop-out.

Recruitment targets will vary across sites based on research capacity and the number of eligible patients.

### 2.3 Study setting

The study is designed to be delivered fully remotely, using a home sleep apnoea test (WatchPAT^®^ 300) and an electronic data capture system (REDCap) for consent and data collection. However, some study procedures may also take place over the telephone or face-to-face (e.g., at the memory clinic or in a participant’s home) to minimise digital exclusion (Wilson-Menzfeld et al., 2025).

### 2.4 Study procedures

Participants will undergo study assessments as shown in Table 2.

**TABLE 2:** Summary of study assessments for the Sleep Apnoea and Memory (SAM) study.

Study assessment	Frequency	Main outcomes
Medical history and screening	Twice (consent, then updated after 6 months or at study end)	History of sleep apnoea; relevant comorbidities and treatments; degree of cognitive impairment and suspected etiology (if known)
Respiratory polygraphy (WatchPAT^®^ 300)	Once	ODI; RDI; pAHI; hypoxic burden;% sleep in supine position;% of sleep exceeding 45dB threshold for snoring; apnoeas in REM and N-REM sleep stages
Participant-led questionnaires (demographics and sleep apnoea questionnaires)	Once	ESS; STOP-Bang; presence of recognised sleep apnoea symptoms; patient characteristics such as age, sex, and ethnicity; family and patient history of sleep apnoea; user experience; support required to complete the study.
Blood tests (bloods sub-study only)	At 4–6 week intervals, before and after treatment	Biomarker analysis including amyloid-beta 40 and 42; phosphorylated tau 181 and 217; glial fibrillary acidic protein; neurofilament light chain.

ODI, oxygen desaturation index; pAHI, peripheral apnoea-hypopnea index, REM, rapid eye movement, RDI, respiratory disturbance index.

#### 2.4.1 Main study

##### 2.4.1.1 Screening and eligibility

Eligibility will be confirmed by a member of the research team. Clinical data on relevant diagnoses, medications, and medical history will be obtained from medical records and/or directly from patients, including information on the most recent cognitive diagnosis, scores from recent cognitive assessments, and neuroimaging or fluid biomarker results where available. Due to the likelihood of new diagnoses or cognitive changes, where available, medical records will be reviewed at 6 months and at the end of the study for any relevant cognitive updates (e.g., new diagnosis, biomarker results).

##### 2.4.1.2 Home sleep apnoea testing

Participants will be asked to complete a single night of respiratory polygraphy using the WatchPAT^®^ 300, a HSAT device recommended by NICE (NG202; NICE, 2021). The WatchPAT^®^ 300 collects data across seven channels from three points of contact (at the fingertip, chest, and wrist): peripheral arterial tone (PAT) signal, heart rate, oximetry, actigraphy, body position, snoring, and chest motion. Earlier versions (WatchPAT^®^ 100 and 200) have been demonstrated to have good sensitivity and acceptable specificity compared to conventional PSG (Moffa et al., 2023) and have been previously used in patients with MCI and AD dementia (Tadokoro et al., 2020).

Participants will also be asked to complete a demographics questionnaire and a questionnaire for symptoms and risk factors of sleep apnoea, including the ESS (Johns, 1991) and STOP-Bang (Chung et al., 2016) alongside bespoke items as recommended by NICE (NICE, 2021). Additional domains covered beyond the ESS and STOP-Bang include subjective sleep quality, feeling refreshed upon awakening, impact of poor sleep on daily functioning, daytime napping, morning headaches, nocturnal sweating, nocturia, nasal congestion, and breathlessness. The questionnaire will also include more general questions around sleep disturbance and include single screening questions for restless legs syndrome and rapid eye movement (REM) sleep behavior disorder (Ferri et al., 2007; Postuma et al., 2012). We will also ask patients to rate how easy or difficult the device was to use, if they have sleep apnoea whether they used any treatment during their polygraphy recording, as well as provide general feedback on their experience (Riha et al., 2023).

The WatchPAT^®^ 300 devices will be provided to participants via the dedicated WatchPAT^®^ Direct service, who will also provide participants with written instructions for use and pre-paid envelopes for return postage (or arrange home collection where necessary). Upon return, the WatchPAT^®^ Direct team will upload the data to a secure cloud-based platform, CloudPAT^®^. Data from the WatchPAT^®^ 300 will be manually reviewed alongside data collected from the medical history and patient-led questionnaires by a sleep physiologist with experience in interpreting polygraphy for sleep apnoea assessments. The participant and their GP will be informed of the study results by the local site team. Standard local procedures will be followed if referrals for further investigation, treatment, or follow-up are indicated.

Where the quality or duration of the recording is insufficient (e.g., shorter than 4 h’ sleep), the data will not be analysed and results will not be fed back, and participants will be invited to complete another night at their discretion. Participants who decline the repeat the recording but agree to continue with other study tasks will not be withdrawn from the study.

#### 2.4.2 Bloods sub-study

At consent, participants at the lead participating site (North Bristol NHS Trust) will be asked whether they agree to be invited to a blood biomarker sub-study if they are referred for sleep apnoea treatment. All participants who consent to this optional sub-study, receive a new diagnosis of sleep apnoea from the main study, and are referred for treatment will be invited to participate in the blood biomarker sub-study.

This exploratory sub-study aims to observe whether there are changes in blood biomarkers relevant to neurodegeneration and AD after sleep apnoea treatment. Participants will be asked to undergo a series of blood tests approximately 4–8 weeks apart, starting as soon as possible after referral (before their appointment with the sleep service) and spanning the period leading up to treatment initiation and for up to 6 months after their treatment has started. Participants will be invited to a maximum of nine blood draws, depending on participant availability and acceptability. Alongside blood draws, we will also collect data on treatment initiation (e.g., treatment type, date) and treatment adherence (Oliver et al., 2022) from the participant and sleep service where available.

Blood plasma will undergo analysis for biomarkers relating to AD and neurodegeneration based on the panels available and recommended for use at the time of analysis. It is anticipated that the Aβ42/40 ratio, phosphorylated tau (p-tau) 181, p-tau 217, glial fibrillary acidic protein, and neurofilament light chain will be the primary biomarker panel (Fu and Ho, 2026). However, in light of the rapidly evolving field of biomarkers, we may also integrate additional biomarkers relating to AD or neurodegeneration if these achieve widespread adoption or clinical validation during the study period (Grigoli et al., 2024). This will give us an indication if treating sleep apnoea may slow the progression of AD and will support proof-of-concept evidence for future, large-scale treatment studies.

### 2.5 Main outcomes

#### 2.5.1 Prevalence of sleep apnoea

The primary outcome will be the proportion of people in memory clinics who have at least mild sleep apnoea as defined by an ODI 3% ≥ 5. We will also report the proportion of people with mild (ODI 3% ≥ 5 < 15), moderate (ODI 3% ≥ 15 < 30), and severe (ODI 3% ≥ 30) sleep apnoea. Sensitivity analyses will assess the proportion of sleep apnoea as defined by ODI 4%, AHI 3%, and AHI 4% cut-offs.

The prevalence of sleep apnoea will also be reported for each etiology (e.g., AD, Lewy body disease) with at least 10 cases.

#### 2.5.2 Predictors of sleep apnoea

The predictive accuracy of the STOP-Bang, ESS, and additional questions for at least mild sleep apnoea will be calculated: sensitivity, specificity, and area under the receiver operating characteristic (ROC) curve.

#### 2.5.3 Feasibility of home sleep apnoea testing

Recruitment and retention will be recorded alongside data completeness and reasons for missingness or withdrawals.

We will record the number of people who opt for the sleep apnoea questionnaire completed online, by telephone, by post, or face-to-face, as well as data completeness for each method. We will also record the proportion of participants who required support at home to engage in the study.

#### 2.5.4 Bloods sub-study

We will report recruitment to the bloods sub-study, treatment initiation and adherence, and change in blood biomarkers over time (before and after treatment).

### 2.6 Patient and public involvement

Patient and public involvement (PPI) representatives with lived experience of cognitive impairment and/or sleep apnoea have been involved since study inception. SB, who has lived experience as a carer for someone living with dementia, is part of the research team, having supported the initial funding and ethics applications, and providing continued oversight of the study to ensure public perspectives are included throughout the full research process. Additional PPI representatives have advised on the relevance of the project and helped to develop the study protocol and patient-facing materials, including advising on wording for the bespoke questions in the sleep apnoea questionnaire. PPI representatives will also be invited to regular meetings with the research team and steering committee.

### 2.7 Data analysis

Descriptive statistics will summarise participant demographics, clinical characteristics, sleep questionnaire responses (e.g., STOP-Bang, ESS) and objective sleep parameters derived from respiratory polygraphy.

For sleep apnoea prevalence outcomes, descriptive statistics will be provided alongside 95% confidence intervals.

Participants currently on treatment for sleep apnoea will not be asked to cease treatment. While our primary outcome is the prevalence of ODI3% >5 (on or off treatment), we will also report the number and proportion of people who report prior diagnosis of sleep apnoea, whether sleep apnoea is already being treated, and whether treatment appears to be successful (ODI3% < 5 on treatment). We will also report the proportion of patients recommended for referral for treatment or further investigation due to sleep apnoea and other incidental findings (e.g., possible periodic limb movements or atrial fibrillation).

For predictors of sleep apnoea, area under the ROC curve will be calculated alongside sensitivity and specificity to individually assess accuracy of the ESS and STOP-Bang as well as individual component scores. Adaptive least absolute shrinkage and selection operator (LASSO) will be used to explore predictive models for sleep apnoea. The proportion of data completeness will be reported for each predictor and outcome variable along with the proportion of complete cases and where available, reasons for missing data. Multiple imputation may be used in prediction model development.

Participant flow will be reported in line with STROBE guidance (Von Elm et al., 2007).

Interrupted time series analysis will be used for blood biomarker analysis, with a time series model fit with linear regression using each biomarker as an outcome including time as a predictor, an indicator variable to represent pre-intervention or postintervention period, and a time by intervention period indicator variable interaction.

## 3 Discussion

This project will provide important insights into the prevalence and symptoms of sleep apnoea within memory clinics. Currently, treatments for patients attending memory clinics are limited. Sleep, and sleep apnoea specifically, can be improved with interventions and may represent a modifiable risk factor for dementia. Identifying the prevalence and symptoms of sleep apnoea in memory clinics and the feasibility of remote testing are important steps toward future clinical trials where we may be able to slow disease progression and/or improve cognitive outcomes and quality of life.

A primary strength of this study is its prospective, multi-center design which will recruit participants from several NHS memory clinics across England. It is likely that estimates of prevalence vary between individual studies and clinics at least in part due to methodological differences in screening (Mubashir et al., 2019) but may also be related to different study populations. We will be able to compare prevalence across different sites, which have been chosen to be geographically and socioeconomically diverse.

Data collection is also patient-centered and decentralised. Individuals can participate entirely remotely if able to, completing respiratory polygraphy and questionnaires at home unsupervised and at a time that is convenient for them. However, participants will also be offered telephone or in-person support to participate in the study as needed.

However, several methodological considerations should be considered. Firstly, there may be participant selection biases. Individuals who subjectively experience potentially relevant symptoms (e.g., daytime sleepiness) or subjective sleep disturbance may perceive benefits to a diagnosis and be more willing to participate in the study. Alternatively, individuals who might be negatively impacted by a sleep apnoea diagnosis (e.g., people who are driving) may be more likely to decline to participate. Individuals may also decline to participate if they anticipate difficulties with study participation, which could overestimate feasibility. Secondly, in the absence of EEG and nasal airflow, HSAT devices cannot provide a PSG-equivalent AHI and may be less accurate in some cases (Campbell and Sulaiman, 2023). Thirdly, prevalence estimates may be affected by the definition of sleep apnoea, and it remains under debate which metrics (e.g., 3% or 4%, ODI or AHI) are most important in prognosis. Finally, whilst the bloods sub-study may provide proof-of-concept, large-scale clinical trials will be needed to identify whether treatment of sleep apnoea is beneficial to brain health in this population.
